# MicroRNAs as biomarkers of brain injury in neonatal encephalopathy: an observational cohort study

**DOI:** 10.1038/s41598-024-57166-z

**Published:** 2024-03-19

**Authors:** Fatima Dakroub, Firas Kobeissy, Stefania Mondello, Zhihui Yang, Haiyan Xu, Livia Sura, Candace Rossignol, Mehmet Albayram, Dhanashree Rajderkar, Kevin Wang, Michael D. Weiss

**Affiliations:** 1 Department of Experimental Pathology, Immunology and Microbiology, Center for Infectious Diseases Research, American University of Beirut, Beirut, Lebanon; 2 Center for Neurotrauma, MultiOmics and Biomarkers, Department of Neurobiology, Morehouse School of Medicine, Atlanta, GA USA; 3 Department of Biomedical and Dental Sciences and Morphofunctional Imaging, University of Messina, Via Consolare Valeria 1, 98125 Messina, Italy; 4 Department of Emergency Medicine, University of Florida, 1149 Newell Drive, L3-166, Gainesville, FL 32611 USA; 5 Department of Pediatrics, University of Florida, 1600 SW Archer Road, Gainesville, FL 32610-0296 USA; 6 Department of Radiology, University of Florida, Gainesville, FL 32610 USA; 7 Brain Rehabilitation Research Center, Malcom Randall VA Medical Center, North Florida/South Georgia Veterans Health System, 1601 SW Archer Road, Gainesville, FL 32608 USA

**Keywords:** Neonatal encephalopathy, HIE, MicroRNA biomarkers, Diagnostic markers, Molecular neuroscience, Neonatal brain damage

## Abstract

Neonatal Encephalopathy (NE) is a major cause of lifelong disability and neurological complications in affected infants. Identifying novel diagnostic biomarkers in this population may assist in predicting MRI injury and differentiate neonates with NE from those with low-cord pH or healthy neonates and may help clinicians make real-time decisions. To compare the microRNA (miRNA) profiles between neonates with NE, healthy controls, and neonates with low cord pH. Moreover, miRNA concentrations were compared to brain injury severity in neonates with NE. This is a retrospective analysis of miRNA profiles from select samples in the biorepository and data registry at the University of Florida Health Gainesville. The Firefly miRNA assay was used to screen a total of 65 neurological miRNA targets in neonates with NE (n = 36), low cord pH (n = 18) and healthy controls (n = 37). Multivariate statistical techniques, including principal component analysis and orthogonal partial least squares discriminant analysis, and miRNA Enrichment Analysis and Annotation were used to identify miRNA markers and their pathobiological relevance. A set of 10 highly influential miRNAs were identified, which were significantly upregulated in the NE group compared to healthy controls. Of these, miR-323a-3p and mir-30e-5p displayed the highest fold change in expression levels. Moreover, miR-34c-5p, miR-491-5p, and miR-346 were significantly higher in the NE group compared to the low cord pH group. Furthermore, several miRNAs were identified that can differentiate between no/mild and moderate/severe injury in the NE group as measured by MRI. MiRNAs represent promising diagnostic and prognostic tools for improving the management of NE.

## Introduction

MicroRNAs (miRNAs) are small endogenous RNA molecules that regulate translation in eukaryotic cells at the post-transcriptional level. They bind to their target mRNAs to induce translational repression or degradation^1–3^. MiRNAs are small, released into the extracellular space, resistant to degradation, and easily detected^3,4^. These attributes highlight their suitability as biomarker candidates for neonatal encephalopathy (NE). Unlike neuroproteins, miRNAs are detectable at very small concentrations. Since several miRNAs are involved in normal brain development, alterations in their expression may be implicated in conditions like NE.

NE is a major cause of lifelong disability in affected infants, causing profound distress for families and driving huge costs for healthcare systems^5^. The clinical management of neonates with NE is complex. Biomarkers capable to inform treatment, monitor injury progression, and predict outcomes are urgently needed. Currently, there is an active effort to develop neuroprotein-based biomarkers to assist the bedside clinician with real-time decision making such as treatment initiation^6–12^. MiRNAs may provide complementary information enabling clinicians to characterize and stratify risk among neonates with NE^13–17^.

This study aims to assess the miRNA profiles in neonates with NE undergoing therapeutic hypothermia and compare them to healthy controls and neonates with a low cord pH. We hypothesized that miRNA expression levels would differentiate between neonates with moderate to severe NE compared to the control and low cord pH populations. Moreover, we aimed to assess miRNA expression in NE neonates who underwent hypothermia according to brain injury severity.

## Materials and methods

### Study design and setting

This is a retrospective observational cohort study of miRNA profiles from select specimens collected between December 2013 and May 2019 and obtained from the Florida Neonatal Neurologic Network registry and biorepository as previously reported^18,19^. The University of Florida Institutional Review Board approved the study (IRB#201501109, IRB201802816).

### Participants

#### Neonatal encephalopathy (NE) group

To identify subjects with NE, data were extracted from the electronic medical records and recorded in the REDCap electronic data capture tools^20^. From each patient in the NE group, two samples were retrieved from the biorepository and analyzed. The first sample was collected 0–6 h after birth, and the second sample was collected 48 h after birth. These timepoints coincided with the time before hypothermia (0–6 h) or at the initiation of hypothermia and during hypothermia (48 h).

Specimens were selected by NE outcome based on magnetic resonance imaging (MRI) results and categorized into no/mild brain injury (n = 18) and moderate/severe injury (n = 18) groups.

#### Low umbilical cord pH with or without evidence of mild NE

The alteration in umbilical cord pH, namely acidosis, reflects hypoxic stress in the fetus. Low cord pH, as an isolated finding, is not a predictor of hypoxic ischemic injury. However, low cord pH combined with other abnormal clinical findings is associated with adverse neonatal outcomes. Hence, we included a sample of 18 neonates with low umbilical cord pH to determine whether their miRNA expression profile differed compared with neonates with NE. The low umbilical cord pH group included neonates with a cord pH ≤ 7.1 who did not develop moderate to severe NE. They were transitioned to the NICU for closer monitoring, collection of clinical labs, and amplitude-integrated electroencephalography (aEEG) monitoring.

Neonates with a normal neurologic exam, normal labs, and/or an aEEG with no evidence of hypoxic-ischemic injury for the six-hour monitoring period were transferred back to the mothers, regardless of the pH. One blood sample was collected at 0–6 h of life from scavenged blood and stored in the biorepository.

#### Control subjects

A total of 37 healthy neonates were included in the study. The eligibility criteria for controls included an Apgar score of eight or higher at one and five minutes of life (i), no evidence of encephalopathy (ii), and admission to the newborn nursery (iii). One blood sample was collected at birth from each patient’s umbilical cord from scavenged blood and later obtained for analysis from the biorepository^18,19^**.** Umbilical arterial samples were targeted. Of the 37 control samples, 25 were labeled as mixed because the arterial samples may have been contaminated by some umbilical venous blood at the time of sampling, five samples were definitively umbilical arterial and six samples were definitively labeled as umbilical venous. A single sample was not labeled.

### MicroRNA profiling

Blood samples (1 ml) were centrifuged at 1200 g for 15 min. The resulting sera were transferred into 2 ml cryovials and stored at − 80 °C until assay analysis. The Firefly miRNA particle assay system was used for miRNA measurement, coupled with a portable flow cytometer/reader (Guava® easyCyte™ 6HT, Millipore, Burlington, MA). RNA extraction was performed by incubating 20 µl serum with 40 µl Lysis Mix and 20 µl nuclease-free water for 45 min at 60 °C while shaking. Then, the extracted RNA was analyzed with the FirePlex™ miRNA neurology panel V2 (cat# ab218371, Abcam, Waltham, MA) as described previously (PMC9301366).

### Magnetic resonance imaging (MRI) scoring

In neonates with NE, MRIs were performed at either 4–5 days of age following rewarming (n = 30) or at 7–12 days of age (n = 6) if the babies were not stable enough for transport to MRI at 4–5 days of age. Neonates were imaged on a 3 T scanner (Siemens, Malvern, PA). The analysis focused on the T1-weighted, T2-weighted, and diffusion-weighted imaging (DWI) abnormalities. Two blinded subspecialty board-certified neuroradiologists with over 10 years of experience interpreted the MRI images using the Barkovich scoring system^21^. Individual brain regions scored included the basal ganglia (0–4), the watershed cortex/white matter (0–5), and combined basal ganglia/watershed (0–4). Infants with scores of 0–2 in any region were categorized as no/mild injury on MRI. Scores ≥ 3 in any region were interpreted as moderate/severe injury. Examples of neonatal brain MRI images showing injury to the basal ganglia and diffuse injury to the cortex and subcortical white matters are presented in Figure S1.

### Data management and statistical analysis

No formal calculation of the sample size was performed as analyses were done retrospectively and included all neonates with NE who fulfilled the inclusion criteria. Baseline characteristics were summarized using standard descriptive statistics. Continuous variables were described as mean and standard deviation (SD), and categorical data were summarized as absolute frequencies and percentages. We compared groups using Mann–Whitney tests (two groups) and Kruskal–Wallis tests with post-hoc Dunn’s test (three groups). The association between categorical variables was evaluated using the chi-square or Fisher’s exact test, as indicated. Multivariate analysis methods were used to identify relevant patient clusters and variables responsible for class discrimination^22,23^. We first used principal component analysis (PCA) to lower the dimensionality of the data and to identify distinct clusters and potential outliers within the data sets. Outliers were identified using score plots in combination with Hotelling’s T2 and distance to model in X-space (DModX)^24,25^. Subsequently, a supervised multivariate analysis was conducted using orthogonal partial least squared discriminant analysis (OPLS-DA). OPLS-DA was used to maximize the detection of miRNA (X-variables) associated with differentiation between the predefined groups. In a subsequent modeling step, the loadings associated with each model were screened for the top 10 relevant variables using the R2VXAdj metric (i.e., explained a fraction of the variation of X variables for the predictive component). Traditional statistical analysis was performed using R (http://www.r-project.org, version 3.5.1) in RStudio (http://www.rstudio.com, version 1.1.456), and SIMCA® 16 Software (Umetrics AB, Umeå, Sweden) was used for multivariate data analysis.

### Sub-network enrichment pathway analysis

To identify the potential pathobiological relevance of the identified miRNA markers that characterize NE we performed miRNA Enrichment Analysis and Annotation (miEAA) using the PathwayStudio software (Elsevier Inc. https://www.elsevier.com/solutions/pathway-studio-biological-research). To determine significantly altered functional and biological pathways for each set of identified miRNAs, the Subnetwork Enrichment Analysis (SNEA) algorithm was utilized. This algorithm uses Fisher’s exact test for the detection of nonrandom associations between two categorical variables that are organized by a specific relationship. Furthermore, the distribution of the subnetwork was compared between the inside and outside of the pathway using the Mann–Whitney test. Pathways with a *P*-value less than 0.05 were considered significant.

### Statement of financial support

Department Development Funding.

### Consent statement

Informed consent was obtained within 72 h of birth from the parents of all neonates enrolled.

### Statement on study methodology

The University of Florida Institutional Review Board approved the study (IRB#201501109, IRB201802816). Informed consent was obtained for sample and data collection for all subjects enrolled in the biorepository. Blood collection from neonates was conducted in conformity with common practice as well as state and federal regulations.

## Results

### Description of the patient population

We studied 36 neonates with moderate to severe NE who underwent hypothermia. The mean gestational age was 38.2 weeks (SD ± 1.9), the mean birth weight was 3383 g (SD ± 867), and the cohort consisted primarily of males (61%). Neonates characteristics at enrollment were analyzed by no/mild and moderate/severe brain injury as assessed by MRI (Table 1). The Apgar scores at 5 and 10 min of life, the Sarnat scores, and the history of seizures were different between both groups (*P* < 0.05). The Apgar scores were lower in the moderate/severe brain injury group, which also included more infants with a stage III initial Sarnat exam and a history of seizures. Additional characteristics and details are shown in Table 1.

**Table 1:** The demographical and clinical characteristics of NE neonates (n = 36).

Infant characteristics at enrollment	NE (n = 18)	NE (n = 18)
	No/mild injury on MRI	Moderate/severe injury on MRI
Sex, n (%)		
Female, n (%)	3 (17)	9 (50)
Male, n (%)	15 (83)	9 (50)
Race, n (%)		
White	10 (56)	11 (61)
Black	4 (22)	4 (22)
Other	4 (22)	3 (17)
Gestational Age, mean (SD), weeks	38.4 (1.8)	38.0 (2.2)
Birth weight, mean (SD), grams	3364 (792)	3402 (958)
Apgar score at 1 min, mean (SD)	2.2 (1.4)	1.4 (1.6)
*Apgar score at 5 min, mean (SD)	5.1 (1.9)	2.8 (2.3)
*Apgar score at 10 min, mean (SD)	6.5 (2.0)	4.3 (2.7)
Sentinel Event, n (%)	4 (22)	9 (50)
C-Section delivery, n (%)	7 (39)	11 (61)
*History of seizures, n (%)	3 (17)	13 (72)
*SARNAT score II, n (%)	17 (90)	8 (50)
*SARNAT score III, n (%)	1 (10)	10 (50)
Initial pH, mean (SD)	7.11 (0.1)	6.9 (0.2)
Initial Base Deficit, mean (SD)	−16.1 (4.3)	−18.7 (8.0)
Initial Lactate, mean (SD)	9.9 (5.6)	12.9 (5.4)

*NE* Neonatal encephalopathy, *SD* standard deviation.

*p-value < 0.05.

Moreover, a total of 37 age-matched healthy controls and 18 neonates with a low cord pH were included in this study. In the low cord pH group, all neonates had Sarnat serial exams performed between 0 and 6 h after birth. Of the 18 neonates, eight had a Sarnat exam consistent with mild NE.

When comparing the low cord pH group (n = 18) to the NE group (n = 36), there were no significant differences in gestational age, birth weight, sex, and Cesarian section (Table S2). However, the Apgar scores at 1 and 5 min of life were higher in the low cord pH group (3.8 ± 3.1 and 7.2 ± 1.8) compared to the NE group (1.80 ± 1.6 and 3.94 ± 2.4) (Table S2, *P* < 0.05).

### Comparison of NE neonates with healthy controls and with low cord pH neonates

MiRNA profiles of neonates with moderate to severe NE who underwent hypothermia were compared to those from healthy controls using PCA (R2X = 0.67, Q2 = 0.60). A degree of natural separation was observed (Figure S2). Supervised OPLS-DA modeling maximized the variations between the two groups (R2Y = 0.91, Q2 = 0.83, *p* < 0.0001) (Figure S2a), with an area under the curve (AUC) of 0.99 (Figure S2b). The loadings plot indicated that different miRNAs characterized the two groups (Figure S2c).

**Figure 1:**
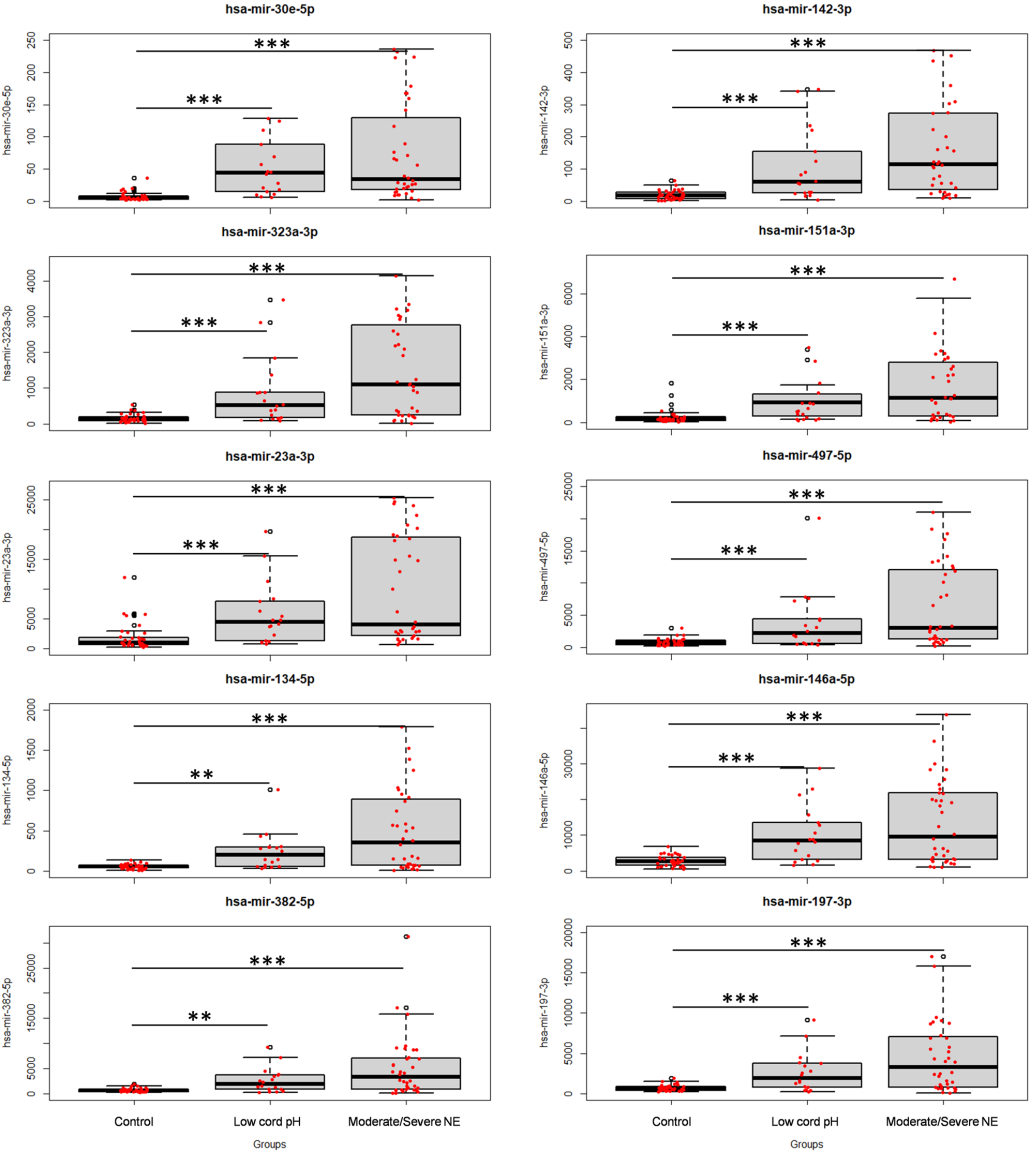
Box-and-whisker plots of highly influential miRNA in healthy, low pH and NE neonates. The group differences are presented for 10 selected miRNAs. The black horizontal line in each box represents the median, with the boxes representing the interquartile range. Each dot represents a patient. Significant differences are indicated with *(*p* < 0.05), **(*p* < 0.01), or ***(*p* < 0.001) (Kruskal–Wallis test).

A set of highly influential miRNAs that can differentiate between the NE group and controls was identified (Fig. 1). All were significantly upregulated in the NE group compared to the control group. Interestingly, the same significant trend was observed when comparing the low cord pH group to healthy controls. The highest fold change in miRNA levels was obtained for miR-323a-3p and mir-30e-5p.

MiRNA profiles of neonates with NE were additionally compared to those from neonates with a low cord pH. An OPLS-DA model could be constructed (R2Y = 0.47, Q2Y = 0.28, *p* = 0.003) (Figure S3a), with the ability to separate the two groups with an area under the ROC curve of 0.91 (Figure S3b). The loadings plot associated with the model was screened for relevant variables (Figure S3c).

**Figure 2:**
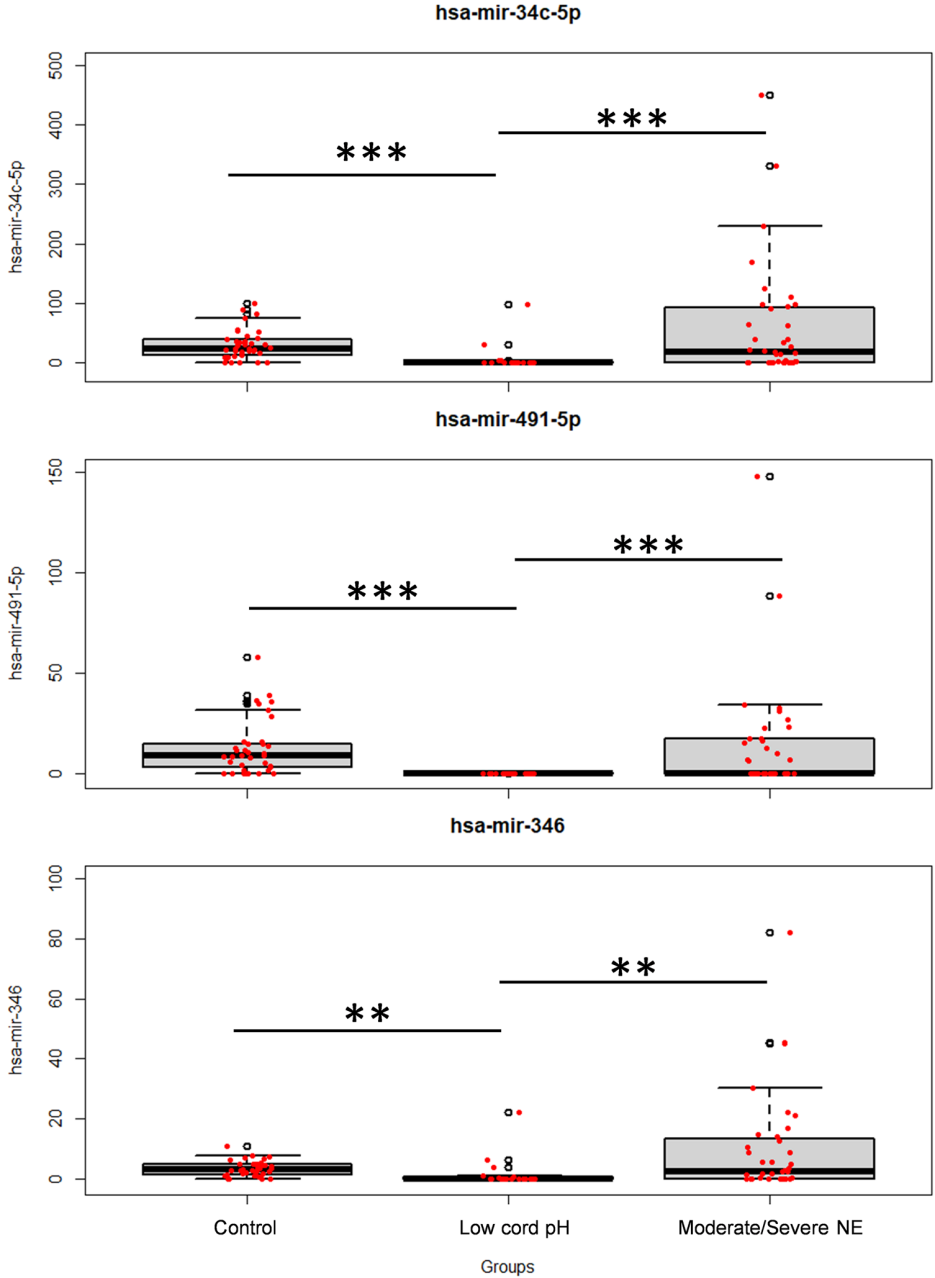
Group differences for identified miRNA discriminating low cord pH and NE groups. Box-and-whisker plots of highly influential miRNA in healthy, low pH and severe HIE neonates. The black horizontal line in each box represents the median, with the boxes representing the interquartile range. Each dot represents a patient. Significant differences are indicated with *(*p* < 0.05), **(*p* < 0.01), or *** (*p* < 0.001) (Kruskal–Wallis test).

In total, three miRNAs were significantly different between low cord pH and moderate to severe NE neonates. The levels of miR-34c-5p, miR-491-5p, and miR-346 were significantly lower in the low cord pH group compared to the moderate to severe NE group and the healthy controls (Fig. 2).

### Comparison of brain injury as assessed by MRI

MiRNA profiles of the neonates with NE were compared according to the severity of brain injury on MRI using multivariate models. The analysis was conducted twice for samples taken before and after hypothermia. OPLS-DA models (Figure S4) could be constructed using baseline characteristics and miRNA profiles obtained before and after hypothermia (R2Y = 0.69, Q2Y = 0.41, *p* = 0.002 and R2Y = 0.58, Q2Y = 0.41, *p* = 0.003, respectively), with ability to separate the two groups with an area under the ROC curve of 0.97 and 0.996, respectively.

**Figure 3:**
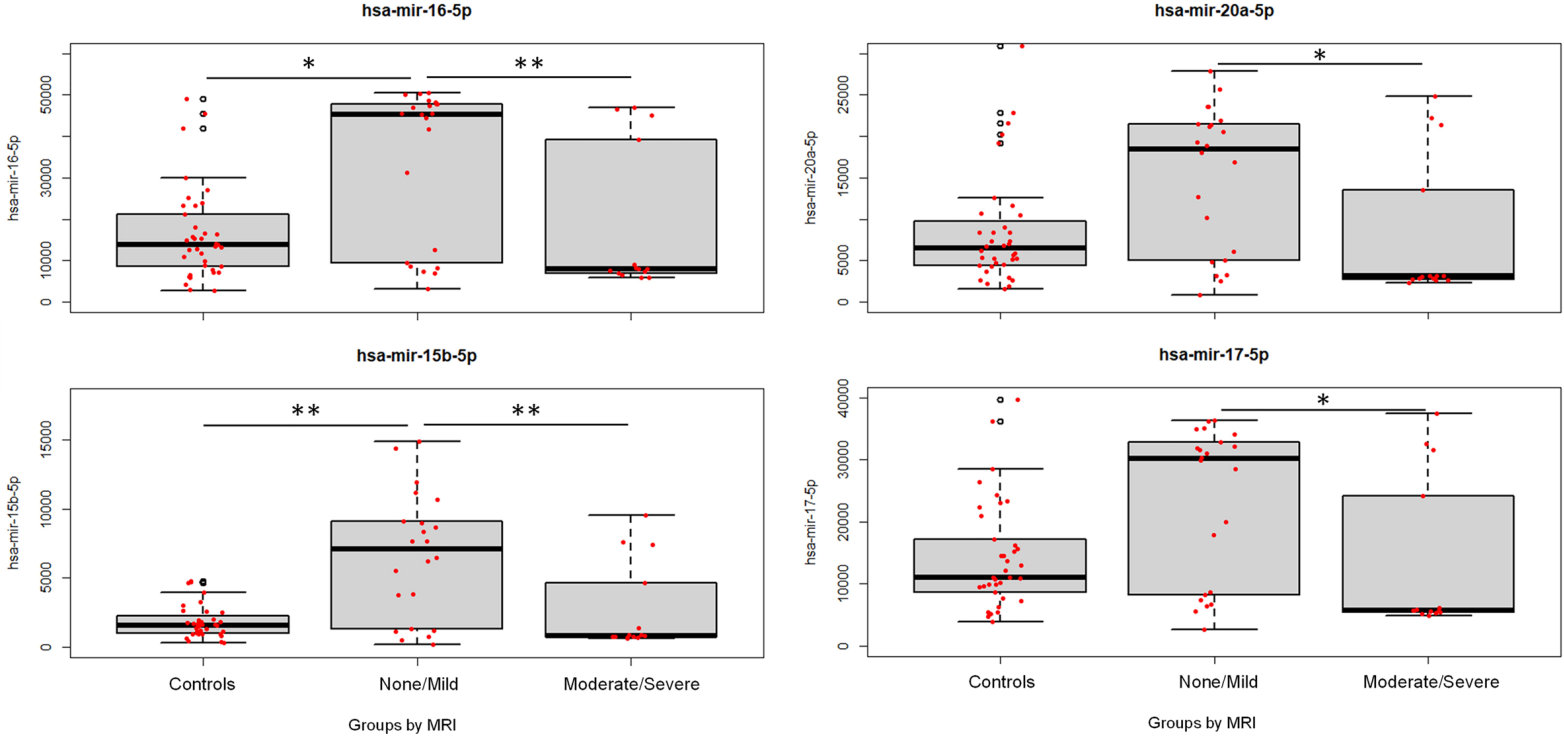
Box-and-whisker plots demonstrating MiRNA expression in blood samples taken before or at initiation of hypothermia (at 0–6 h post-birth) from NE neonates with moderate/severe injury and with no/mild injury on MRI, and in controls. The black horizontal line in each box represents the median, with the boxes representing the interquartile range. Significant differences are indicated with*(*p* < 0.05) or with **(*P* < 0.01) (Kruskal–Wallis test).

The loading plots revealed that the moderate/severe injury group is characterized by a higher occurrence of seizures (*p* < 0.0001), increased AST and ALT levels (*p* = 0.007), and a lower APGAR score at 5 min (*p* = 0.02) compared to the no/mild injury group. Hsa-mir-16-5p (*p* = 0.006), hsa-mir-20a-5p (*p* = 0.02), hsa-mir-15b-5p (*p* = 0.01), and hsa-mir-17-5p (*p* = 0.01) were significantly higherin the no/mild brain injury group compared to the moderate/severe injury group and the control groupacutely before or at the time of initiation of hypothermia (Fig. 3).

**Figure 4:**
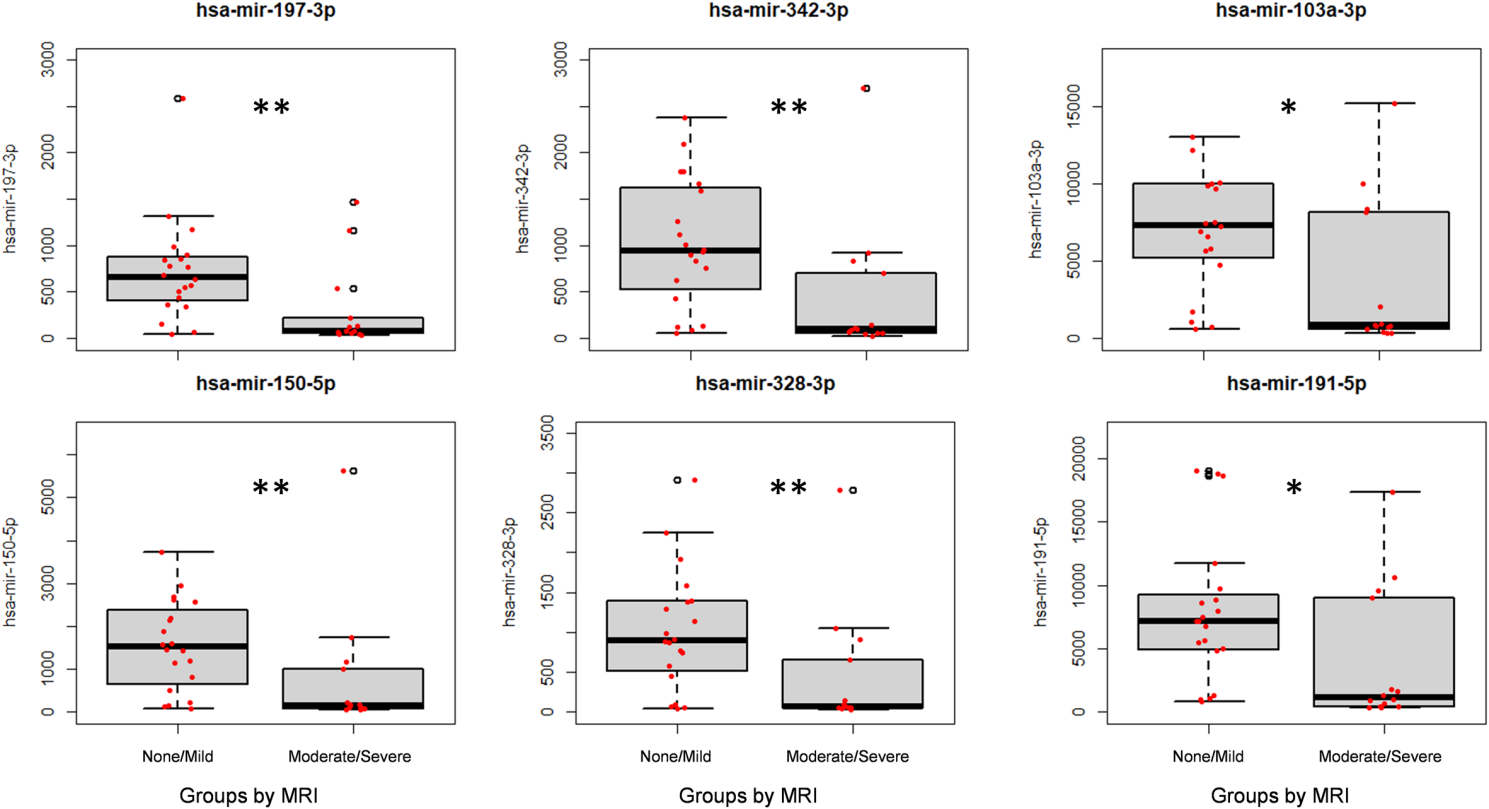
Box-and-whisker plots demonstrating MiRNA expression in blood samples taken after hypothermia (at 48 h post-birth) from NE neonates with moderate/severe injury and with no/mild injury on MRI. The black horizontal line in each box represents the median, with the boxes representing the interquartile range. Significant differences are indicated with*(*p* < 0.05) with or **(*P* < 0.01) (Kruskal–Wallis test).

Subacutely at 48 h, during hypothermia, we demonstrated that the expression of hsa-mir-197-3p (*p* = 0.004), hsa-mir-342-3p (*p* = 0.003), hsa-mir-103a-3p (*p* = 0.01), hsa-mir-150-5p (*p* = 0.006), hsa-mir-328-3p (*p* = 0.0096) and hsa-mir-191-5p (*p* = 0.03) was significantly reduced in the moderate/severe injury group (Fig. 4).

## Discussion

MiRNA expression affects a plethora of biological pathways implicated in ischemic injury, including apoptosis and neuroinflammation. Investigating their role as selective biomarkers of NE is an impressive research field that has the potential to improve risk stratification in such a heterogeneous injury.

The first aim of this study was to determine miRNAs that are significantly altered within hours of life in neonates suffering from NE compared to healthy controls. The expression levels of 10 miRNAs were significantly upregulated in the NE group compared to healthy controls. The biological pathways in which these miRNAs are implicated are presented in Figure S5. Casey et al. utilized a porcine model of neonatal NE to demonstrate a significant increase in circulating miR-151a levels one-hour post-hypoxia–ischemia^26^. Interestingly, miR-151-a was shown to promote healing after spinal cord injury by targeting P53 to improve neuronal survival^27^. In response to injury, microglia release exosomes containing miR-151-a that can be taken up by neurons to attenuate cell death. This may explain the pronounced upregulation of miR-151-a-3p in NE neonates in this study. Similarly, the upregulation of miR-146a-5p in NE neonates may serve as a counteractive mechanism against neuroinflammation and oxidative stress. MiR-146a is a known negative regulator of inflammation that inhibits the interleukin-1 receptor-associated kinase 1 (IRAK1) and toll-like receptor 4 (TLR4) genes in immune cells^28^. Analysis utilizing the Pathway Studio software additionally predicts the role of miR-146a in the inhibition of ischemia and neuroinflammation (Figure S5). These data suggest that miR-151-a and MiR-146a may be upregulated as a defense mechanism to ameliorate portions of the pathophysiologic cascade following hypoxia–ischemia^11^.

Only a few studies exist comparing the miRNA concentrations between neonates with NE and those with low umbilical cord pH^29^. We found that in neonates with low cord pH, blood concentrations of miR34c-5p, miR491-5p, and miR346 were substantially lower than in moderate to severe NE and healthy controls. These results suggest distinct pathobiological processes underlying low cord pH and its consequences, but not NE. This is also supported by the lack of differences between low cord pH and control groups. Further studies are warranted to fully understand these findings.

Next, we investigated miRNA profile alterations in NE neonates requiring therapeutic hypothermia according to MRI brain injury severity. Analysis revealed a significant upregulation in the levels of miR-16-5p, miR-15b-5p, miR-17-5p, and miR-20a-5p in patients with no/mild injury at 0–6 h of age compared to moderate/severe injury group and controls. These miRNAs are implicated in the regulation of cell proliferation and apoptosis. Previous studies have reported that miR-16-5p and miR-15b-5p induce apoptosis by targeting the ani-apoptotic protein BCL-2^33–35^. Pathway studio analyses predict that miR-15b-5p is involved in apoptosis regulation, while miR-16-5p positively regulates apoptosis (Figure S5). The overexpression of miR-15b-5p and miR-16b-5p induces cell cycle arrest in G1-G0, and their levels negatively correlate with those of cyclin D1^36^. The dynamic injury landscape of neonatal NE and the various time points post-injury are two factors that influence the type of cell death implicated in the disease. Following acute neonatal HI injury, strikingly necrosis is the major cell death phenotype in the brain of mouse, rat, and piglet neonates^37–39^. Moreover, necrosis is associated with early neurodegeneration after HI, while apoptosis is associated with delayed neuronal death. This may explain the upregulation of apoptosis-associated miRNAs in the no/mild injury group compared to moderate/severe injury group, and the lack of difference between healthy controls and moderate/severe injury group. While, these findings should be interpreted with caution because the limited sample size and the inherent heterogeinity of NE, they provide initial evidence of the role of apoptosis as a major component of the pathophysiologic cascade in no/mild brain injury, and suggest that miR-16-5p and miR-15b-5p have potential to identify subjects most likely to respond to therapeutic hypothermia by ameloriating apoptosis^40^, and be used as predictive markers and for drug development in the moderate to severe injury group.

MiR-17-5p and miR-20a-5p were shown to prevent cell cycle arrest during neuronal lineage differentiation of stem cells^41^. miEAA predicts that they are implicated in the regulation of cell proliferation and differentiation (Figure S5). Interestingly, the overexpression of the miR-17–92 cluster upregulates cell proliferation in ischemic animals^42^. Branyan et al. showed that stroke outcomes were improved when miR-20a-3p was administered by intravenous injections 4 h after middle cerebral artery occlusion (MCAo) in rats^43^. MiR-17-5p and miR-20a-5p were upregulated in the no/mild injury group compared to controls, probably as a an attempt to mitigate the pathological effects of hypoxia. This increase is not observed in the moderate/severe injury group. Clinically, this calls for the investigation of miR-17-5p and miR-20a-5p as attractive candidates for future NE treatment. Moreover, these markers may affect synaptic plasticity and long-term outcomes and merit further study.

In this study, we have also measured miRNAs at 48 h during hypothermia to identify potential markers of treatment response, and foud that miR-197-3p, miR-342-3p, miR-328-3p, miR-191-5p, miR-103a-3p, and miR-150-5p were significantly upregulated in patients with no/mild injury compared to the moderate/severe group and controls. It was previously established that miR-150-5p promotes cell survival to protect against ischemic injury in the heart^44^. Additionally, the neuroprotective role of miR-150-5p was revealed in the hypoxic-ischemic brain^45^. Scherrer et al. reported that lower log-miR-150-5p levels in the plasma were associated with mortality three months post-ischemic stroke^46^. Clinically, this finding reflects the potential of miR-150-5p as an attractive marker for monitoring the therapeutic response following hypothermia.

Of the neonates with seizures (36%) in our cohort, 85% had EEG-confirmed seizures and 15% had clinical seizures. Our study demonstrated that AST, ALT, and the presence of seizures were associated with moderate/severe brain injury on MRI. Seizures have been reported to correlate with worse outcomes in neonates with NE^47^. These observations are not novel, but they demonstrate that our cohort is consistent with others reported in the literature^47,48^. Our results highlight the association between miRNA levels and the potential mechanisms of liver injury, as well as seizure activity in the injured neonatal brain.

One of the strong points of this study is the inclusion of matched healthy controls and a considerable number of neonates with NE compared to previous studies^15,49,50^. Moreover, we assessed miRNA profile alterations in the low cord pH group. This allowed us to identify miRNAs that can differentiate between neonates with low cord pH and those with NE, suggesting that these two conditions are associated with distinct molecular mechanisms. Furthermore, we utilized sera to allow the assessment of only extracellular miRNA. We offered a practical approach for NE diagnosis by utilizing circulating markers which are convenient and efficient.

One limitation of neonatal biomarker studies is the lack of studying only neonates with sentinel events, which occur in 15–29% of NE cases^51,52^. These events include placental abruptions, uterine ruptures, and shoulder dystocia accompanied by the interruption of placental function or the disruption of fetal blood flow and leading to hypoxia–ischemia in the fetus. Unlike adults with traumatic brain injury or stroke, the exact timing of injury in NE may be unknown. Sentinel events allow for the exact timing of the hypoxic-ischemic insult. We performed a sub-group analysis of all neonates with sentinel events, but the sample size was small. Additionally, we analyzed MRI results by subjective reading. Ideally, objective readings with volumetric measurement of the injury should be used to correlate injury with the serum concentrations of biomarkers. Finally, control and low cord pH samples were obtained at birth and 1 h of age respectively. While these were ideal controls for the 0–6 h of age NE group, we were unable to obtain control and low cord pH samples at 48 h of age and therefore comparisons at this time point may not be ideal.

In conclusion, this study provides pilot data that confirm the alteration of miRNA profiles of neonates with moderate to severe NE compared to healthy controls. There is limited research on miRNA in the context of NE. Our results can be utilized to focus the research efforts on each identified miRNA marker individually. Moreover, the identified miRNAs in this study have the potential for utilization as quantifiable noninvasive biomarkers for NE diagnosis and therapeutic response monitoring.

## Supplementary Information

Supplementary Information.

## Data Availability

The datasets generated and/or analysed during the current study are available from the corresponding author on reasonable request.

## Abbreviations

ABG: Arterial blood gas
aEEG: Amplitude-integrated electroencephalography
AUC: Area under the curve
CK: Creatine kinase
CK-MB: Creatine kinase-myoglobin binding
DWI: Diffusion-weighted imaging
HIE: Hypoxic-ischemic encephalopathy
IRAK: Interleukin-1 receptor-associated kinase 1
LFT: Liver function test
MCAo: Middle cerebral artery occlusion
miEAA: MiRNA Enrichment Analysis and Annotation
miRNAs: MicroRNAs
MRI: Magnetic resonance imaging
NE: Neonatal encephalopathy
NICU: Neonatal Intensive Care Unit
OPLS-DA: Orthogonal partial least squared discriminant analysis
PCA: Principal component analysis
PTT: Partial thromboplastin time
SNEA: Subnetwork Enrichment Analysis
SST: Serum separator tubes
TBI: Traumatic brain injury
TLR4: Toll-like receptor 4
