# Supplementary material for: MicroRNAs as biomarkers of brain injury in neonatal encephalopathy: an observational cohort study
Source: Sci Rep. 2024 Mar 19;14:6645. doi: 10.1038/s41598-024-57166-z (PMC10951356; doi:10.1038/s41598-024-57166-z)
Supplement: Supplementary file 1 — Supplementary Information. [file 41598_2024_57166_MOESM1_ESM.docx]

**Table S1. The demographic and clinical characteristics of neonates belonging to the NE (n=36) and low cord pH groups (n=18).**

| *p-value <0.05 | **Low cord pH (n=18)** | **NE Group (n=36)** |
| --- | --- | --- |
| **Gestational age in weeks (mean ± SD)** | 38.7±1.49 | 38.22±1.97 |
| **Male sex n (%)** | 12 (67%) | 22 (61%) |
| **Birth weight in grams (mean ± SD)** | 3419±516 | 3383±867 |
| ***Outborn n (%)** | 0 | 13 (36%) |
| **C-section mode of delivery n (%)** | 10 (56%) | 18 (50%) |
| ***APGAR at 1 and 5 min (mean ± SD)** | 3.8 ± 3.14  7.2 ± 1.80 | 1.80 ± 1.57  3.94 ± 2.40 |
| **Initial pH (mean ± SD)** | 7.07 ± 0.05 | 7.04 ± 0.2 |
| **Initial Base Deficit (mean ± SD)** | -11.3 ± 3.4 | -15.7 ± 9.96 |
| ***Initial Lactate (mean ± SD)** | 5.5 ± 4.9 | 11.4 ± 5.7 |

*MRI, Magnetic resonance imaging; NE, Neonatal encephalopathy; SD, Standard deviation; *p-value <0.05*

*
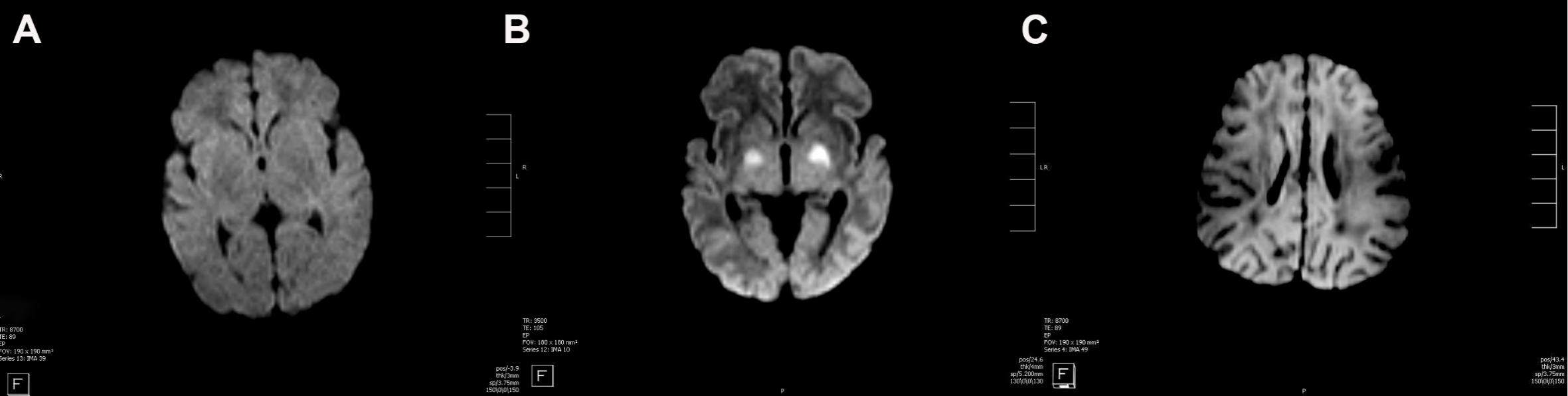

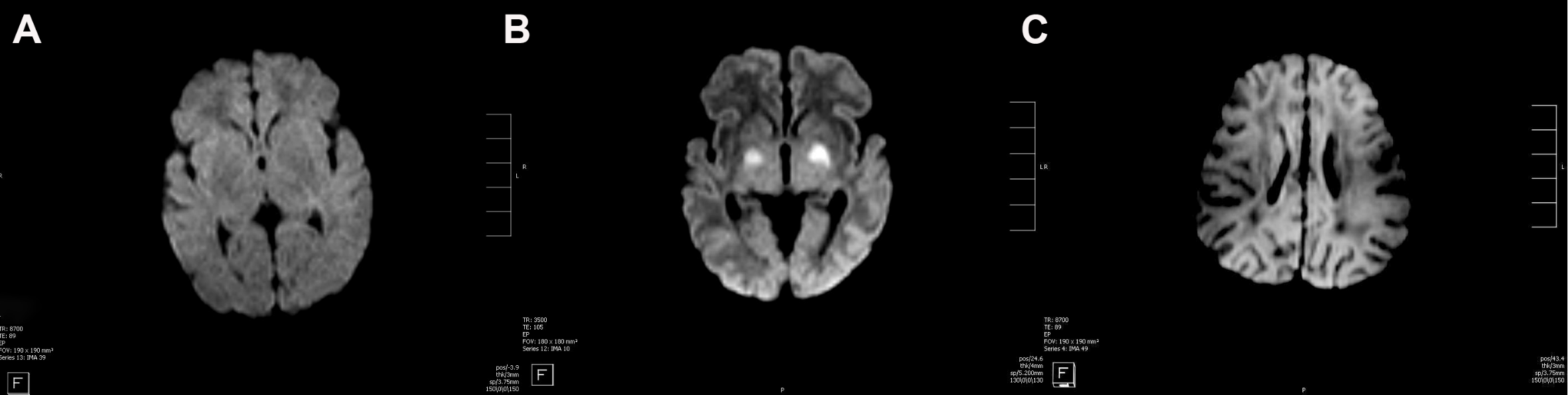
*

*
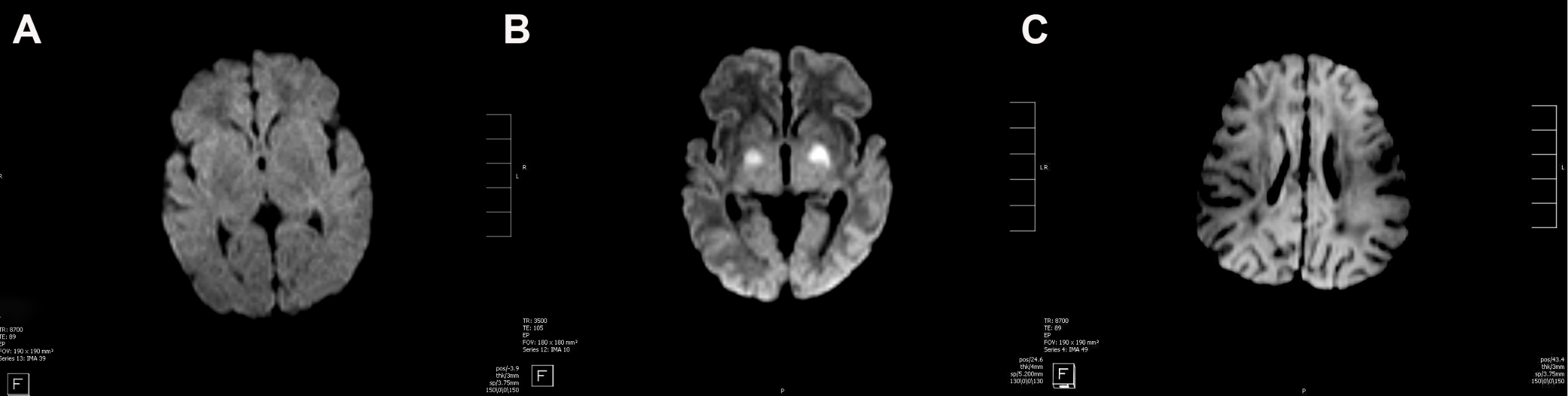
*

**Figure S1.  Diffusion weighted MRI images of the brain in a neonate with no injury (A), injury to the basal ganglia (B) and diffuse injury to the cortex and subcortical white matter (C).**

**c.**

**b.**

**Figure S2: Neonatal miRNA profiles in the NE group and healthy control groups (CTR). (a) OPLS-DA scores plot** **comparing miRNA profiles from neonates with moderate to severe NE who underwent hypothermia (red circles) and healthy controls (green circles). Each dot represents a patient. (b) ROC curve for the cross validated scores from the OPLS-DA model (AUC 0.99). (c) Loading plot of the OPLS-DA model, showing how the miRNAs (X-variables) relate to each other as well as to group belonging (Y-variables).**


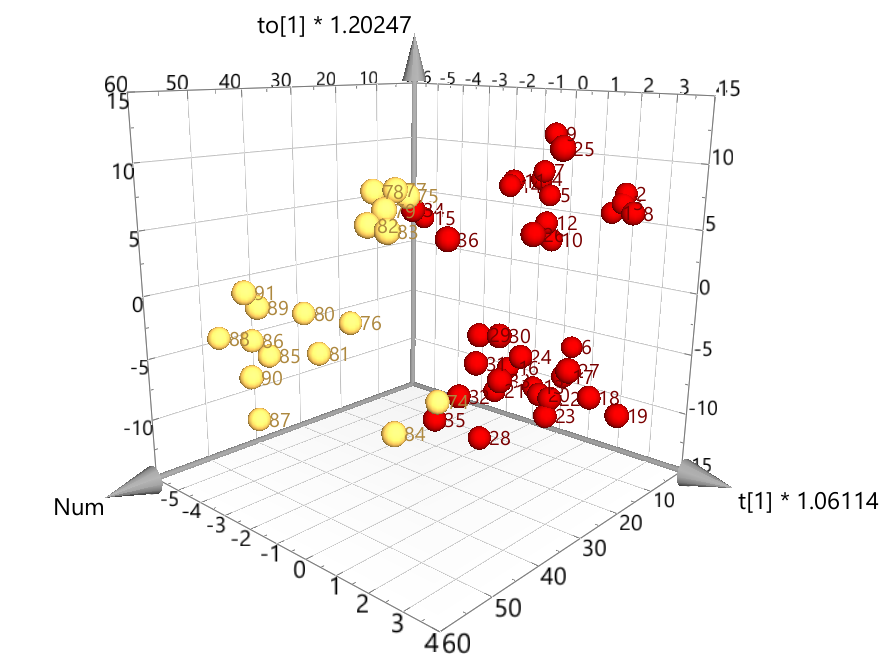


**b.**

**a.**

**c.**

**Fig.S3. a) OPLS-DA scores plot of circulating MiRNA data between neonates with NE (red circles) and low cord pH (yellow circles). Each dot represents a patient. b) Loading plot of the OPLS-DA model, showing how the miRNAs (X-variables) relate to each other as well as to group belonging (Y-variables). c) ROC curve of the OPLS-DA analysis between moderate-to-severe HIE and low cord pH neonate (AUC 0.90).**


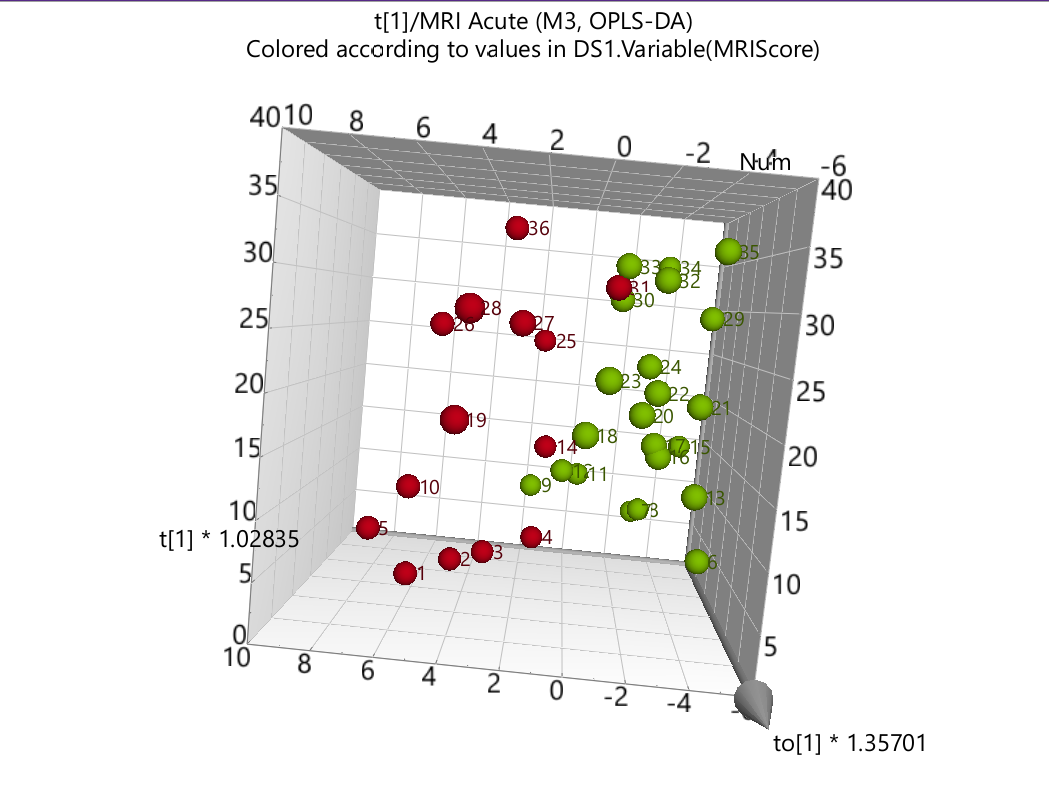


**c.**

**b.**

**a.**

**d.**


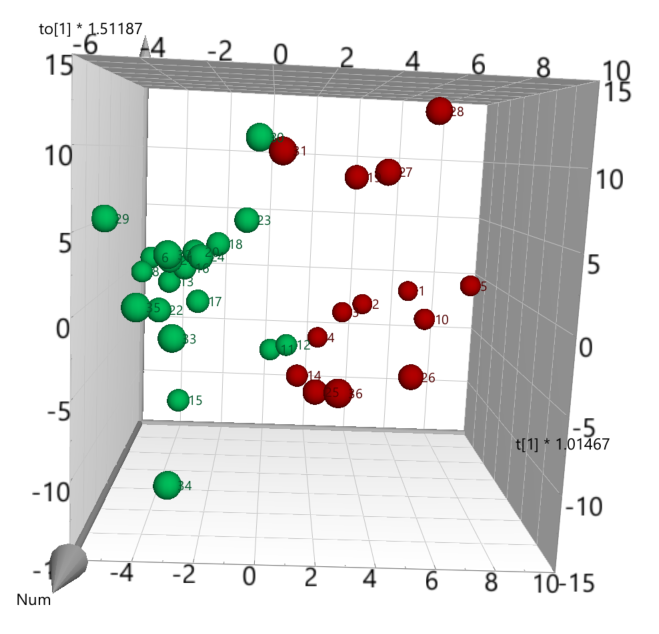


**Fig.S4. OPLS-DA scores plot with one orthogonal and one aligned component, comparing samples taken a) before hypothermia c) and after hypothermia from NE neonates who developed moderate/severe injury on MRI (red circles) and those with no/mild injury on MRI (green circles). ROC curves of the OPLS-DA analyses - AUC 0.97 b) and 0.996 d).**

**
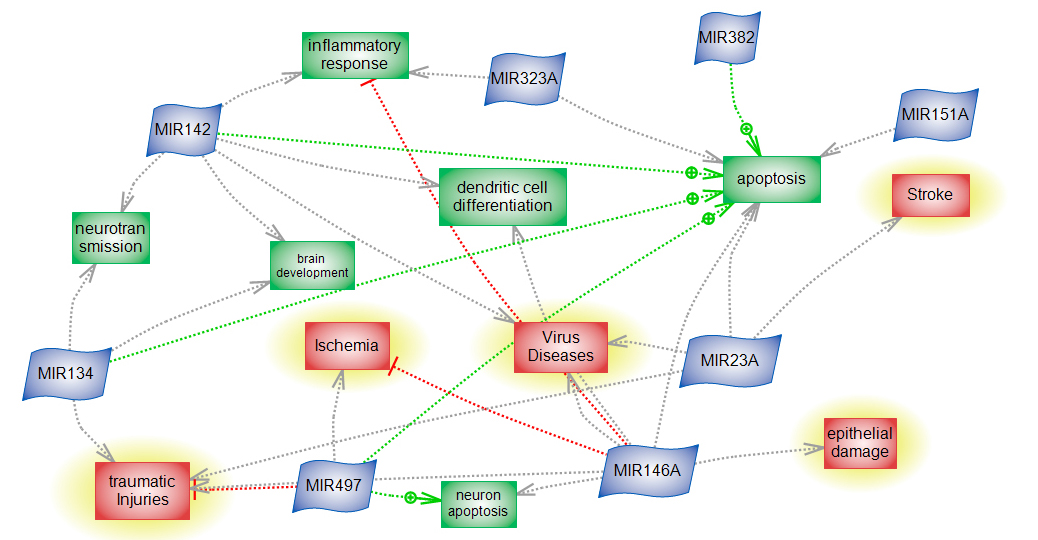
**

**Fig. S5. The biological pathways regulated by the identified highly influential miRNA in healthy, low pH and NE neonates.**

**
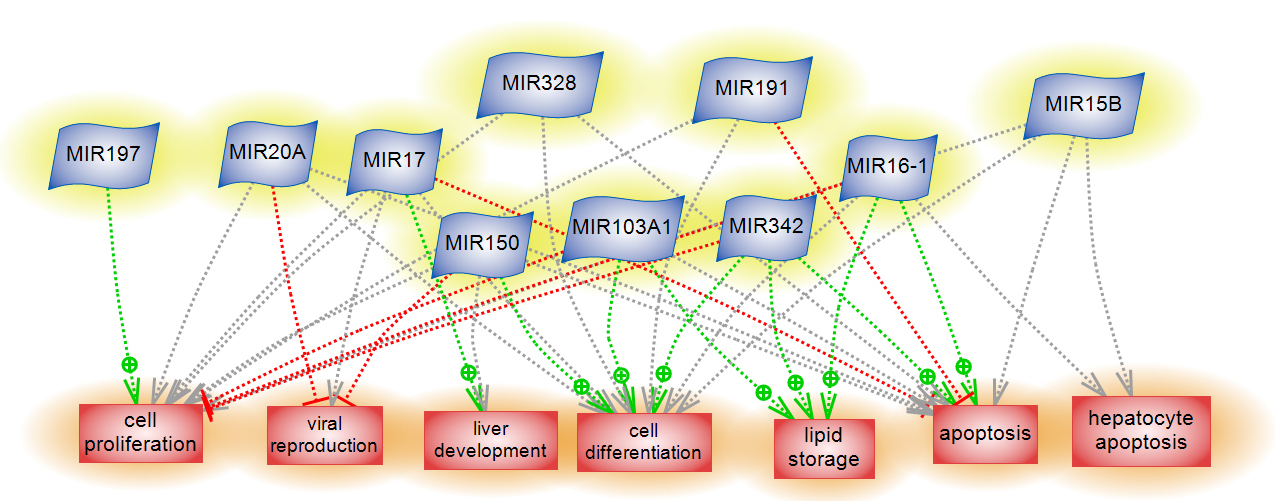
**

**Fig. S6. The biological pathways regulated by the miRNAs that differentiate between moderate/severe and no/mild brain injury in the NE group.**
